# Genome-wide expression profiling of the response to short-term exposure to fluconazole in *Cryptococcus neoformans *serotype A

**DOI:** 10.1186/1471-2180-11-97

**Published:** 2011-05-11

**Authors:** Ada Rita Florio, Selene Ferrari, Elena De Carolis, Riccardo Torelli, Giovanni Fadda, Maurizio Sanguinetti, Dominique Sanglard, Brunella Posteraro

**Affiliations:** 1 Istituto di Microbiologia, Università Cattolica del Sacro Cuore, Largo F. Vito 1, 00168 Rome, Italy; 2 Institute of Microbiology, University of Lausanne and University Hospital Center, Lausanne, Rue du Bugnon 48, CH-1011 Lausanne, Switzerland

## Abstract

**Background:**

Fluconazole (FLC), a triazole antifungal drug, is widely used for the maintenance therapy of cryptococcal meningoencephalitis, the most common opportunistic infection in AIDS patients. In this study, we examined changes in the gene expression profile of the *C. neoformans *reference strain H99 (serotype A) following FLC treatment in order to investigate the adaptive cellular responses to drug stress.

**Results:**

Simultaneous analysis of over 6823 transcripts revealed that 476 genes were responsive to FLC. As expected up-regulation of genes involved in ergosterol biosynthesis was observed, including the azole target gene *ERG11 *and *ERG13*, *ERG1*, *ERG7*, *ERG25*, *ERG2*, *ERG3 *and *ERG5*. In addition, *SRE1 *which is a gene encoding a well-known regulator of sterol homeostasis in *C. neoformans *was up-regulated. Several other genes such as those involved in a variety of important cellular processes (i.e. lipid and fatty acid metabolism, cell wall maintenance, stress and virulence) were found to be up-regulated in response to FLC treatment. Conversely, expression of *AFR1*, the major transporter of azoles in *C. neoformans*, was not regulated by FLC.

**Conclusions:**

Short-term exposure of *C. neoformans *to FLC resulted in a complex altered gene expression profile. Some of the observed changes could represent specific adaptive responses to the antifungal agent in this pathogenic yeast.

## Background

*Cryptococcus neoformans *is a basidiomycetous fungal pathogen that causes meningoencephalitis in predominantly immunocompromised hosts [1,2], that is the most devastating manifestation of cryptococcal disease and is fatal unless treated [3]. Cryptococcosis appears to be a significant opportunistic infection in solid-organ transplant recipients, with a prevalence rate ranging from 0.26% to 5% and overall mortality of 42% [4]. Notably, cryptococcal meningitis was reported to occur in 46% of patients from an Indian HIV-positive cohort [5]. Although the introduction of highly active antiretroviral therapy has led to a decrease in the number of cryptococcal infections in AIDS patients in most developed countries, this is not the case in developing countries where the incidence of HIV/AIDS and cryptococcal meningitis continue to rise [6]. As fluconazole (FLC) became increasingly used due to the need for life-long maintenance therapy in HIV/AIDS patients, FLC resistance was hence detected at relatively high frequency in *C. neoformans *clinical isolates from India, Africa and Cambodia [7-9].

Increased FLC resistance *in vitro *was shown to be predictive of treatment failures and infection relapses [10]. Recently, the mechanism underlying the heteroresistance to FLC was elucidated [11], that is an adaptive mode of azole resistance previously associated with FLC therapy failure cases [12]. This mechanism is based on duplications of multiple chromosomes in response to drug pressure [13]. Interestingly, Sionov et al. [13] observed that the number of disomic chromosomes positively correlated with the duration of exposure to FLC, whereas the duplication of chromosome 1 was closely associated with two genes, *ERG11*, the target of FLC [14], and *AFR1*, the major transporter of azoles in *C. neoformans *[11,15]. Such genomic plasticity enables cells to cope with drug stress and was observed in *C. neoformans *strains of both serotypes, A (*C. neoformans *var. *grubii*) and D (*C. neoformans *var. *neoformans*) [13].

The recent sequencing of the *C. neoformans *genome [16] has stimulated the development of *C. neoformans*-specific microarrays that made possible to address hypotheses about global responses to overcome stresses during growth in the human host [17,18]. Regardless of the source (i.e. host-derived or antifungal drugs), toxic compounds exert constant selective pressure on the fungus that responds by developing mechanisms necessary for survival [19].

With the aim to identify genes required for adaptive growth in the presence of sub-inhibitory concentrations of FLC, we investigated here the transient response of *C. neoformans *to FLC by analyzing differences in gene expression prior and after FLC exposure of strain H99, a reference strain of serotype A. Thus, genome-wide transcriptional profiling of over 6823 *C. neoformans *genes identified 476 genes with significant expression changes. Apart from genes involved in ergosterol biosynthesis (e.g. *ERG11*), genes involved in other important cellular functions, such as those encoding the sterol homeostasis regulator Sre1 [20] or phospholipase B1 (Plb1) [21], were shown to be induced by FLC treatment. In addition, *AFR1 *was not found FLC-responsive, suggesting indirectly that this gene is responsible for long-term FLC adaptation in *C. neoformans*.

## Methods

### Strain, growth conditions and RNA isolation

*C. neoformans *var. *grubii *serotype A strain (H99) was obtained from David S. Perlin [22], kept as 20% glycerol stock at -80°C and sub-cultured, as required, on YEPD (1% yeast extract, 2% peptone, 2% glucose) agar plates at 30°C. For RNA isolation independent overnight cultures were diluted 1:100 in liquid YEPD and grown at 30°C or 37°C with agitation for 3 h to reach a density of 3 × 10^7 ^CFU/ml. At this point cultures were equally divided into two aliquots to which either FLC at a concentration of 10 mg/l or distilled water was added, followed by incubation at 30°C or 37°C for 90 min. After this treatment, cultures were centrifuged at 4°C and 5500 × *g *and total RNA was extracted as previously described [23].

### Microarray design and preparation

*C. neoformans *H99 microarrays were designed following the Agilent Array Design guidelines (Earray platform) by first creating two separate sets of 60-base nucleotide probes for each of 6967 open reading frame (ORF) sequences as downloaded from the Broad Institute website http://www.broadinstitute.org/annotation/genome/cryptococcusneoformans/MultiHome.html. The probe selection was performed using the GE Probe Design Tool; probes were filtered following their base composition and distribution, cross-hybridization potential, and melting temperature, to yield final duplicate probes representing 6823 ORFs to cover 97.9% of the whole *C. neoformans *H99 genome. *C. neoformans *custom arrays were manufactured in the 8 × 15k format by Agilent Technologies (Santa Clara, CA, USA). For quality control and normalization purposes, 157 probes were selected randomly and spotted 10 times throughout each array. Standard controls (Agilent Technologies) were also included.

### cRNA synthesis, labeling and hybridization

RNA sample preparation was performed on three biological triplicates of H99 cells grown at 30°C, as described above. Prior to the labeling/amplification step, purity and integrity of the RNA samples were determined using Agilent RNA 6000 Nano LabChip kit on the Agilent 2100 bioanalyzer (Agilent Technologies). Agilent's One-Color Quick Amp Labeling kit (Agilent Technologies) was used to generate fluorescently labeled cRNA probes according to the manufacturer's instructions. The method uses T7 RNA polymerase, which simultaneously amplifies target material and incorporates cyanine 3-labeled CTP. The labeled cRNAs were purified with the RNeasy Mini kit (Qiagen, Hilden, Germany) and quantified using NanoDrop ND-1000 UV-VIS spectrophotometer. Aliquots (600 ng) of Cy3-labeled cRNAs were fragmented and hybridized for 17 h at 65°C to each array using the Gene Expression Hybridization kit (Agilent Technologies) and according to the manufacturer's instructions.

### Microarray imaging and data analysis

Slides were washed and processed according to the Agilent 60-mer Oligo Microarray Processing protocol and scanned on a Agilent microarray scanner G2565BA (Agilent Technologies). Data were extracted from the images with Feature Extraction (FE) software (Agilent Technologies). FE software flags outlier features, and detects and removes spatial gradients and local backgrounds. Data were normalized using a combined rank consistency filtering with LOWESS intensity normalization. The gene expression values obtained from FE software were imported into GeneSpring 10.0.2 software (Agilent Technologies) for pre-processing and data analysis. For inter-array comparisons, a linear scaling of the data was performed using the 75th percentile signal value of all of non-control probes on the microarray to normalize one-colour signal values. Probe sets with a signal intensity value below the 20th percentile were considered as absent and discarded from subsequent analysis. The expression of each gene was normalized by its median expression across all samples. Genes were included in the final data set if their expression changed by at least twofold between strain H99 FLC-exposed or -not exposed (control sample) in at least two independent experiments, together with a *P*-value cut-off of < 0.05 (by one-way analysis of variance [ANOVA] corrected). Genes listed in Table 1 were categorized by reported or putative functions by the BROAD Institute database with NCBI blastP http://www.ncbi.nlm.nih.gov/BLAST/ editing, and also by the Uniprot http://www.uniprot.org/ and *Saccharomyces *genome http://www.yeastgenome.org/cgi-bin/blast-sgd.pl databases. As indicated in Table 1, each *S. cerevisiae *gene name was assigned by blastP search with the *C. neoformans *H99 gene sequence (*e*-value cutoff: e^-6^) according to Kim et al. [24]. Gene Ontology (GO) term analysis was carried to help categorize a list of genes into functional groups. The whole microarray data have been deposited in National Center for Biotechnology Information's Gene Expression Omnibus [25] and are accessible through GEO Series accession number GSE24927.

**Table 1:** Changes in the gene expression of *C. neoformans *H99 cells exposed to FLC

BROAD ID (CNAG_*****)	*C. n*. gene name	*S. c*. gene name	Description	Fold change
Ergosterol biosynthesis				
04804	*SRE1*		Sterol regulatory element-binding protein 1	+ 4.04
01737		*ERG25*	C-4 methyl sterol oxidase	+ 3.95
00854		*ERG2*	C-8 sterol isomerase	+ 3.47
02896		*ERG13*	Hydroxymethylglutaryl-CoA synthase	+ 3.03
06644		*ERG5*	C-22 sterol desaturase	+ 2.50
00040	*ERG11*	*ERG11*	Lanosterol 14 alpha-demethylase	+ 2.47
06829		*ERG1*	Squalene monooxygenase	+ 2.37
00519		*ERG3*	C-5 sterol desaturase	+ 2.21
01129		*ERG7*	Lanosterol synthase	+ 2.09
				
Transport				
04632		*FUR4*	Uracil permease	+ 5.87
07448		*DUR3*	Urea transporter	+ 4.78
04758		*MEP2*/*AMP2*	Ammonium transporter	+ 3.78
06652		*DAL5*	Allantoate permease	+ 2.83
01742		*AQY1*	Water channel	+ 2.73
07902		*CAN1*	Amino acid transporter	+ 2.52
01960		*YMR279C*	Efflux protein EncT	+ 2.47
06338		*PDR15*	ABC transporter PMR5	+ 2.37
04898		*ATR1*	MFS transporter	+ 2.37
00284		*YOR378W*	Efflux protein EncT	+ 2.36
00097		*ITR1*	ITR1	+ 2.26
00895		*ZRT1*	Low-affinity zinc ion transporter	+ 2.20
04210		*MPH2*	Sugar transporter	+ 2.15
04617		*OPT2*	Small oligopeptide transporter	+ 2.11
05592		*PMR1*	Calcium-transporting ATPase	+ 2.06
01059		*YBR241C*	Vacuolar membrane protein	+ 2.02
00904		*AZR1*	Aflatoxin efflux pump AFLT	- 2.10
01769		*AGC1*	Mitochondrial inner membrane protein	- 2.16
04142		*FEN2*	Tartrate transporter	- 2.17
04567		*TPO2*	Drug transporter	- 2.22
05387		*HXT5*	Galactose transporter	- 2.28
02355		*YEA4*	UDP-N-acetylglucosamine transporter	- 2.30
05994		*FLR1*	Multidrug transporter	- 2.35
02733		*STL1*	Hexose transport-related protein	- 2.46
03794		*YBR287W*	Endoplasmic reticulum protein	- 2.58
00815		*SIT1*	Siderochrome-iron (Ferrioxamine) uptake transporter	- 2.92
01354		*TNA1*	Transporter	- 3.39
02104	*SFH5*	*SFH5*	Phosphatidylinositol transfer protein SFH5	- 4.54
07695		*UGA4*	Gamma-aminobutyric acid transporter	- 5.16
00749		*YIL166C*	Transporter	- 5.65
02083		*ARN2*	Siderochrome-iron transporter	- 9.48
				
Cell wall maintenance				
02217		*CHS7*	Chitin synthase 7	+ 3.62
06336		*BGL2*	Glucan 1,3 beta-glucosidase protein	+ 2.61
03326		*CHS2*	Chitin synthase 2, CHS2	+ 2.20
01239	*CDA3*	*CDA2*	Chitin deacetylase	- 4.35
				
Capsule biosynthesis				
03644	*CAS3*		CAS3p	+ 12.16
01489	*CAS9*	*YJL218W*	Putative O-acetyl transferase	- 3.84
				
Lipid and fatty acid metabolism				
06085	*PLB1*	*PLB1*	Phospholipase B	+ 2.18
06623	*MIOX*		Myo-inositol oxygenase	+ 2.12
03128		*ECM38*	Lincomycin-condensing protein lmbA	- 2.01
00424		*PCT1*	Choline-phosphate cytidylyltransferase	- 2.02
05042		*CAT2*	Carnitine acetyltransferase	- 2.10
02000		*FOX2*	Short-chain dehydrogenase	- 2.95
00834		*PSD2*	Phosphatidylserine decarboxylase	- 3.10
02968	*PLC2*		Phospholipase C-2	- 4.11
				
Cell stress				
03400		*GRE2*	Oxidoreductase	+ 3.54
05256		*CTA1*	Catalase 2	+ 2.81
02440		*HSC82*	Cation-transporting ATPase	+ 2.54
01750	*HSP70*	*SSA1*	Heat shock protein 70	+ 2.48
06917	*TSA3*	*PRX1*	Thiol-specific antioxidant protein 3	+ 2.09
03185		*LOT6*	Low temperature-responsive protein	+ 2.05
04622		*SNG1*	Response to drug-related protein	- 2.17
00575		*CTT1*	Catalase	- 2.21
01464	*FHB1*	*YHB1*	Flavo-haemoglobin	- 2.32
				
Amino acid metabolism				
02284		*PDA1*	Branched-chain alpha-keto acid dehydrogenase E1-alpha subunit	+ 2.42
04862		*GLT1*	Glutamate synthase (NADH)	+ 2.39
04017		*MXR2*	Protein-methionine-R-oxide reductase	+ 2.32
01231		*CAR1*	Arginase	+ 2.27
03828		*ARO8*	Aromatic amino acid aminotransferase I	+ 2.26
06540		*ILV3*	Dihydroxy-acid dehydratase	+ 2.18
00247		*LYS9*	Saccharopine dehydrogenase (NADP+, L-glutamate-forming)	+ 2.02
02270		*MET2*	Homoserine O-acetyltransferase	- 2.11
01076		*UGA1*	4-aminobutyrate transaminase	- 2.18
00237		*LEU1*	3-isopropylmalate dehydratase	- 2.27
01264		*LYS12*	Isocitrate dehydrogenase	- 2.31
00879		*GDH2*	Glutamate dehydrogenase	- 2.33
04467		*UGA2*	Succinate-semialdehyde dehydrogenase (NAD(P)+)	- 2.83
02851		*GLY1*	Threonine aldolase	- 3.04
02049		*PUT1*	Proline dehydrogenase	- 5.74
05602		*PUT2*	1-pyrroline-5-carboxylate dehydrogenase	- 6.65
				
Carbohydrate metabolism				
06374		*MAE1*	Malic enzyme	+ 6.04
02225	*CELC*	*EXG1*	Cellulase	+ 3.99
02552		*TKL1*	Transketolase	+ 3.28
04025		*TAL1*	Transaldolase	+ 3.00
00696		*AMS1*	Alpha-mannosidase	+ 2.52
05913		*MAL12*	Alpha-glucosidase	+ 2.34
05113		*ALD4*	Aldehyde dehydrogenase (ALDDH)	+ 2.11
05264		*YJL216C*	Alpha-amylase AmyA	+ 2.08
03946		*GAL1*	Galactokinase	- 2.16
07752	*GLF*		UDP-galactopyranose mutase	- 2.23
04659		*PDC1*	Pyruvate decarboxylase	- 2.33
06924		*SUC2*	Beta-fructofuranosidase	- 2.57
00269		*SOR1*	Sorbitol dehydrogenase	- 2.62
00393	*GLC3*	*GLC3*	1,4-alpha-glucan-branching enzyme	- 2.93
07745	*MPD1*	*ADH3*	Mannitol-1-phosphate dehydrogenase	- 3.54
04217		*PCK1*	Phosphoenolpyruvate carboxykinase	- 8.67
04621		*GSY1*	Glycogen (Starch) synthase	- 11.00
04523		*TDH3*	Glyceraldehyde-3-phosphate dehydrogenase	- 11.45
				
Protein biosynthesis, modification, transport, and degradation				
02389		*YPK1*	AGC-group protein kinase	+ 3.04
02531		*FUS3*	Mitogen-activated protein kinase CPK1	+ 2.91
03176		*ERO1*	Endoplasmic oxidoreductin 1	+ 2.36
05932	*CPR6*	*CPR6*	Peptidyl-prolyl cis-trans isomerase D	+ 2.35
01861		*NAS6*	Proteolysis and peptidolysis-related protein	+ 2.35
04635		*PEP4*	Endopeptidase	+ 2.31
06872		*YKL215C*	5-oxoprolinase	+ 2.27
05005	*ATG1*	*ATG1*	Serine/threonine-protein kinase ATG1	+ 2.20
00919		*KEX1*	Carboxypeptidase D	+ 2.13
04625		*PRB1*	Serine-type endopeptidase	- 2.01
00130		*RCK2*	Serine/threonine-protein kinase	- 2.12
04108		*PKP1*	Kinase	- 2.17
02327		*YFR006W*	Prolidase	- 2.28
02418		*DED81*	Asparagine-tRNA ligase	- 2.40
03563		*DPS1*	Aspartate-tRNA ligase	- 2.50
04275		*OMA1*	Metalloendopeptidase	- 2.50
02006		*NTA1*	Protein N-terminal asparagine amidohydrolase	- 2.75
03949		*PHO13*	4-nitrophenylphosphatase	- 3.32
				
TCA cycle				
03596		*KGD2*	2-oxoglutarate metabolism-related protein	- 2.02
03920		*IDP1*	Isocitrate dehydrogenase (NADP+)	- 2.06
03674		*KGD1*	Oxoglutarate dehydrogenase (Succinyl-transferring)	- 2.52
00747		*LSC2*	Succinate-CoA ligase (ADP-forming)	- 2.70
07363		*IDH2*	Isocitrate dehydrogenase	- 2.80
01137		*ACO1*	Aconitase	- 2.99
07851		*IDH1*	Isocitrate dehydrogenase (NAD+), putative	- 3.80
				
Glycerol metabolism				
06132		*RHR2*	Glycerol-1-phosphatase	+ 2.31
02815		*GUT2*	Glycerol-3-phosphate dehydrogenase	- 2.00
				
Nucleotide metabolism				
05545		*HNT2*	Nucleoside-triphosphatase	+ 2.25
03078		*NPP1*	Type I phosphodiesterase/nucleotide pyrophosphatase family protein	+ 2.08
06489		*ADO1*	Adenosine kinase	- 2.08
00613		*FCY1*	Cytosine deaminase	- 2.69
				
Thiamin metabolism				
03592		*THI20*	Phosphomethylpyrimidine kinase	- 2.51
				
Alcohol metabolism				
05258	*SMG1*		Glucose-methanol-choline (GMC) oxidoreductase	+ 6.67
05024		*SPS19*	L-xylulose reductase	+ 2.53
06168	*GNO1*	*SFA1*	GSNO reductase	- 2.02
				
Carbon utilization				
05144	*CAN2*	*NCE103*	Carbonic anhydrase 2	- 3.18
				
Cell cycle control				
03385		*PCL1*	G1/s-specific cyclin pcl1 (Cyclin hcs26)	+ 2.37
02604		*HOP1*	Putative uncharacterized protein	+ 2.19
00995		*MSC1*	Meiotic recombination-related protein	- 3.63
				
Chromatin and chromosome structures				
02115		*NHP6B*	Nonhistone protein 6	- 2.47
				
Transcription				
01841		*GLN3*	Predicted protein	+ 5.72
02990		*YOR052C*	Nucleus protein	+ 2.16
04594		*UGA3*	PRO1 protein	- 2.01
05290		*SPT3*	Transcription cofactor	- 2.01
06495		*RNH70*	Ribonuclease H	- 2.06
05333		*PUT3*	Putative uncharacterized protein	- 2.14
02338		*GIS2*	DNA-binding protein hexbp	- 2.47
05479		*ASG1*	Putative uncharacterized protein	- 3.57
				
Signal transduction				
03316		*RDI1*	Rho GDP-dissociation inhibitor 1	+ 2.07
00363	*HHK5*	*SLN1*	CnHHK5 protein	- 2.44
01262	*GPB1*	*STE4*	G-protein beta subunit GPB1	- 2.55
Oxidoreduction				
04652		*YLR460C*	Enoyl reductase	+ 2.63
06035		*ADH1*	Alcohol dehydrogenase	+ 2.41
00605		*ZTA1*	Cytoplasm protein	+ 2.20
00038		*SOR2*	Alcohol dehydrogenase	+ 2.13
01954		*YPR127W*	Aldo/keto reductase	+ 2.09
02958		*FET5*	Ferroxidase	+ 2.06
02935		*YMR226C*	Oxidoreductase	- 2.01
01558		*XYL2*	Zinc-binding dehydrogenase	- 2.28
00876		*FRE7*	Ferric-chelate reductase	- 2.49
03168		*MET10*	Sulfite reductase (NADPH)	- 2.55
07862		*YEL047C*	Fumarate reductase (NADH)	- 2.58
03498		*FRE2*	Metalloreductase	- 2.85
03874		*AIF1*	Oxidoreductase	- 2.89
				
Other				
00331		*YMR210W*	Anon-23da protein	+ 3.43
04934	*TAR1*		Temperature associated repressor	+ 2.37
05678		*ADY2*	Membrane protein	+ 2.28
00818		*AGE2*	AGD15	+ 2.23
04867		*YJR054W*	Vacuole protein	+ 2.22
06574	*APP1*		Antiphagocytic protein 1	+ 2.21
06482		*AMD2*	Amidase	+ 2.20
01252		*TUM1*	Thiosulfate sulfurtransferase	- 2.05
03452		*AFG1*	AFG1 family mitochondrial ATPase	- 2.16
05831		*MMF1*	Brt1	- 2.19
03991		*YGR149W*	Integral to membrane protein	- 2.39
02039		*YPL264C*	Integral membrane protein	- 2.46
02943		*SLM1*	Cytoplasm protein	- 2.49
06668		*AIM38*	Mitochondrion protein	- 2.61
00638		*LSG1*	GTPase	- 2.89
01653	*CIG*		Cytokine inducing-glycoprotein	- 3.26
04314		*YEF1*	NAD+ kinase	- 3.74
04690		*FMP41*	Mitochondrion protein	- 5.52

Genes that were found to be differentially expressed were ordered by expression level and categorized, if available, into functional groups as described in Materials and Methods. Results are presented as the mean fold-increase (symbol +) or -decrease (symbol -) of biological triplicates. Abbreviations: *C. n*., *C. neoformans*; *S. c*., *S. cerevisiae*.

### Quantitative RT-PCR (qRT-PCR) validation of gene expression

Expression of selected differentially regulated genes as identified by the microarray analysis was quantitatively assessed with qRT-PCR in an i-Cycler iQ system (Bio-Rad Laboratories, Hercules, CA, USA). All primers and probes (see Additional file 1) were designed with Beacon Designer 2 (version 2.06) software (Premier Biosoft International, Palo Alto, CA, USA) and synthesized by MWG Biotech (Florence, Italy). qRT-PCRs were carried out as previously described [23]. The annealing temperature used for all primers was 65°C. Each reaction was run in triplicate on three separate occasions. For relative quantification of target gene expression, *ACT1 *was used as a normalizer gene [23]. Changes (*n*-fold) in gene expression relative to that of the control were determined from mean *ACT1*-normalized expression levels.

### Oxidative stress and cell wall inhibitor assays

Susceptibilities to hydrogen peroxide (H_2_O_2_) and cell wall inhibitors were measured with exponentially growing cells in liquid YEPD at 30°C or 37°C pre-treated or not with FLC (10 mg/l) for 90 min as described elsewhere with modifications [26,27]. The cells were next washed with sterile PBS and diluted to an OD_650 _of 1.0 in PBS. For the oxidative stress assays, aliquots of the cell suspensions were transferred to Eppendorf tubes where H_2_O_2 _(Sigma, Milan, Italy) was added to 20 mM and incubated at 30°C or 37°C for 2 h. Viability was determined after appropriate dilution of the samples with PBS by plating 100 μl in triplicate on solid YEPD. The CFU were counted after incubation for 72 h at 30°C or 37°C. For the cell wall inhibitor assays, dilutions of the cell suspensions were made in PBS and 5 μl of these were grown on YEPD plates containing 0.5% Congo red (Sigma, C-6767), 0.5, 1.0 and 1.5 mg ml^-1 ^calcofluor white (Sigma, F-3543), 0.01%, 0.03% and 0.06% SDS (Sigma) and 0.2, 0.5 and 1.0 mg ml^-1 ^caffeine (Sigma, C-0750). Plates were incubated for 48 h at 30°C or 37°C and photographed.

## Results and Discussion

### Experimental design and global gene expression results

The transcript profiles of *C. neoformans *H99 cells exposed to 10 mg/l of FLC (1/2 × MIC) for one doubling time (90 min) at 30°C were compared with profiles of untreated cells. A total of 476 genes were found responsive to FLC treatment under the test conditions, consisting of a single concentration and a single time point as described elsewhere [28-30]. The threshold value used in the present analysis was at least a twofold difference of gene expression between the experimental conditions, which is a value generally accepted in fungal genome-wide expression profiling [31]. Given that approximately 95% of the genes (6434/6823) spotted on the microarrays gave validated data, the above mentioned number indicate that 7.4% of the total number of genes in the *C. neoformans *H99 genome exhibited transcriptional changes, with 231 genes being upregulated and 245 downregulated upon FLC treatment.

**Figure 1:**
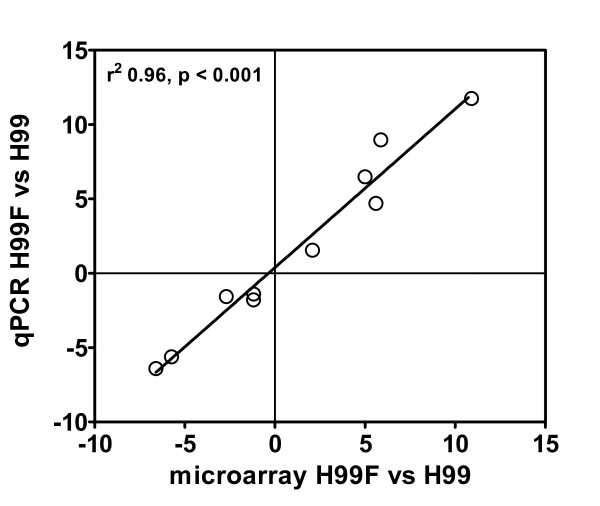
**Scatter plot of the results by microarray and quantitative RT-PCR analyses for ten selected differentially regulated genes in H99 cells FLC-treated (H99F) compared to untreated control cells**.

**Figure 2:**
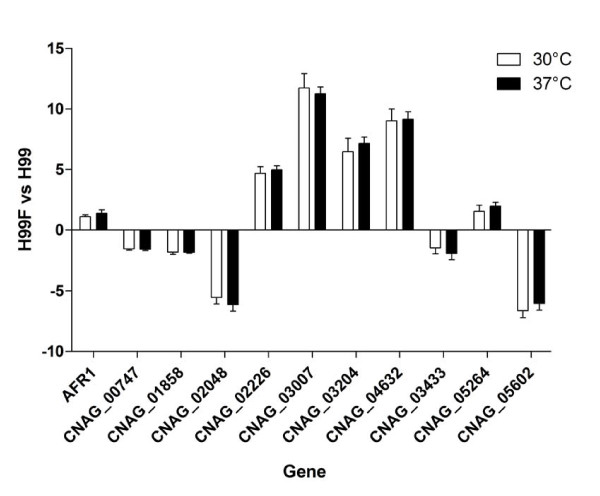
**Results of qRT-PCR analysis performed with RNAs extracted from H99 cells FLC-treated (H99F) at 30°C and 37°C**. The values, which are means of three separated experiments, represent the increase in gene expression relative to untreated control cells (set at 1.00). Error bars show standard deviations

In order to verify the changes in gene expression identified by our microarray analysis, we randomly selected 10 target genes (CNAG_00747, CNAG_01858, CNAG_02048, CNAG_02226, CNAG_03007, CNAG_03204, CNAG_04632, CNAG_03433, CNAG_05264, CNAG_05602) including those regulated and not regulated by FLC for validation of microarray data. A strong correlation (r = 0.94) was found between relative expression levels obtained by microarray or qRT-PCR analysis (Figure 1). In addition, qRT-PCR experiments performed with RNA extracted from H99 cells FLC-treated at 37°C demonstrated that expression of the target genes also including *AFR1 *was comparable to that obtained when H99 cells were pre-treated with FLC at 30°C (Figure 2).

The genes listed in Table 1 were categorized in 10 main groups by functional profiles as described in Methods. The category with the largest number of genes was "transport" with 31 genes, followed by categories that include genes (*n *= 18) involved in carbohydrate metabolism or protein processes (i.e. biosynthesis, modification, transport and degradation). While up- or down-regulated genes were distributed homogenously within almost all the function groups, some categories included more up-regulated genes (ergosterol biosynthesis) or down-regulated genes (TCA cycle). As it will be discussed below, the finding of a large number of genes differentially regulated adds support to the concept that azole activity is beyond the inhibition of the lanosterol demethylase target encoded by *ERG11 *[32], whose overexpression has been associated with fungal resistance [33]. To further classify the genes regulated by FLC exposure, we performed GO term analysis. As expected, GO analysis of genes induced by FLC revealed a significant enrichment of genes involved in sterol metabolism, particularly ergosterol biosynthetic process (Table 2). Enrichment of genes repressed by FLC was observed in processes involving metabolism of amino acids and derivatives (Table 2).

**Table 2:** Gene Ontology (GO) term analysis for the *C. neoformans *FLC response

GO group	GO subgroup	*P*-value
*Up-regulated genes*		
Oxidation reduction		5.26e^-10^
Small molecule metabolic process		1.34e^-06^
	Alcohol metabolic process	4.74e^-07^
	Sterol metabolic process	4.41e^-07^
Steroid metabolic process		7.81e^-07^
	Phytosteroid metabolic process	1.47e^-09^
	Steroid biosynthetic process	9.08e^-07^
	Ergosterol biosynthetic process	3.57e^-08^
Transmembrane transport		0.00076

*Down-regulated genes*		
Oxidation reduction		1.31e^-12^
Small molecule metabolic process		2.50e^-11^
	Alcohol metabolic process	0.00037
	Cellular ketone metabolic process	1.25e^-08^
	Cellular amino acid and derivative metabolic process	3.74e^-12^
	Organic acid metabolic process	1.63e^-08^
Amine metabolic process		1.47e^-13^
	Gamma-aminobutyric acid metabolic process	0.00078

GO term assignment for *C. neoformans *H99 genes was based on homology to *S. cerevisiae *genes. *P*-value represents the probability that a particular GO term is enriched in the microarray gene list. The *P*-value cut-off was < 0.05.

### Effect of FLC on genes involved in ergosterol biosynthesis and related pathways

Earlier efforts to profile the response of yeast cells (*S. cerevisiae *or *C. albicans*) to the short-term exposure to azole drugs implicated genes in the ergosterol biosynthetic pathway as major players [28,29], thus indicating that this pathway is the target of azoles and is responsive to modulations in ergosterol levels. As shown in Table 1, we found that eight *ERG *genes (*ERG1*, *ERG2*, *ERG3*, *ERG5*, *ERG7*, *ERG11*, *ERG13 *and *ERG25*) exhibited increases in expression (2.09- to 3.95-fold) upon FLC treatment. This was a predictable result from the inhibition of Erg11 function by FLC, which is the rate-limiting step of the ergosterol biosynthetic pathway. Indeed, the idea of a compensatory response to re-establish the plasma membrane ergosterol levels [30] may account for the observed upregulation of either early (*ERG13*, *ERG7 *and *ERG1*) or late (*ERG25*, *ERG2*, *ERG3 *and *ERG5*) genes of the ergosterol pathway, in addition to upregulation of *ERG11 *itself (Table 1, *ergosterol biosynthesis*).

*ERG13 *encodes the enzyme hydroxymethylglutaryl-CoA synthase that catalyzes the production of hydroxymethylglutaryl-CoA from acetyl-CoA and acetoacetyl-CoA, and acts in the mevalonate biosynthesis, a precursor required for the biosynthesis of ergosterol. Acetyl-CoA is converted to carbon dioxide and water by enzymes (e.g. isocitrate dehydrogenase) that function in the TCA cycle, a central metabolic process in the mitochondria leading to produce, after oxidative phosphorylation, chemical energy in the form of ATP and NADH. Presumably, as a result of feedback control, we observed that several TCA cycle enzymes were downregulated in response to FLC (Table 1, *TCA cycle*), suggesting that *C. neoformans *may direct the cellular acetyl-CoA content to lipid (sterol) biosynthesis and metabolism to counterbalance ergosterol alteration.

Our particular interest was the up-regulation (4.04-fold) of *SRE1*, that belongs to a group of sterol regulatory element-binding proteins (SREBPs), first characterized in mammalian cells as regulator of lipid homeostasis [34]. While *C. neoformans *Sre1 regulates genes encoding ergosterol biosynthetic enzymes, *SRE1 *was shown to be required for growth and survival in the presence of azoles and also for virulence in a mouse model of cryptococcosis [18,20,35]. In addition, *C. neoformans *Sre1 stimulates ergosterol production in response to sterol depletion when the oxygen-dependent ergosterol synthesis is limited by hypoxia [36]. Consistently, *C. neoformans *mutants in the SREBP pathway showed reduction in ergosterol levels, increased sensitivity not only to low oxygen but also to several chemical agents, including azole antifungals, CoCl_2 _and reactive oxygen species (ROS)-generating compounds. Most importantly, these mutants showed reduced virulence in mice [37].

### Effect of FLC on genes involved in cell structure and maintenance

Consequent to depletion of ergosterol and the concomitant accumulation of 14-methylated sterols, several plausible hypotheses on the mode of action of azoles were suggested by Vanden Bossche [32] two decades ago including alterations in membrane functions, synthesis and activity of membrane-bound enzymes, mitochondrial activities and uncoordinated activation of chitin synthesis. Transcript levels of several genes involving lipid and fatty acid metabolism decreased in the current study (Table 1), possibly in agreement with a remodelling of the cell membrane in response to reduced ergosterol levels. Conversely, expression of *PLB1*, that encodes Plb1, a known virulence factor in *C. neoformans*, was increased 2.18-fold. Phospholipases cleave fatty acid moieties from larger lipid molecules, releasing arachidonic acid for the production of eicosanoids that are utilized by the pathogenic yeasts *C. neoformans *and *C. albicans *to produce immunomodulatory prostaglandins [38]. In addition, cell wall-linked cryptococcal Plb1 contributes to cell wall integrity and is a source of secreted enzyme [39].

It was also expected that exposure to FLC would affect genes responsible for cell wall integrity. Two chitin synthase genes were found to be significantly up-regulated (2.20-fold for *CHS2 *and 3.62-fold for *CHS7*), concomitantly with down-regulated expression (4.35-fold) of the chitin deacetylase *CDA3 *(homolog to *S. cerevisiae CDA2*) (Table 1, *cell wall maintenance*). In *C. albicans*, activation of chitin synthesis, which is mediated by the PKC-, Ca^2+^/calcineurin-, and HOG- cell wall signalling pathways, appears to be an adaptive response to caspofungin treatment. Hence, subculturing caspofungin-resistant cells in the absence of caspofungin resulted in wild-type levels of chitin content [40]. While this form of drug tolerance is rationally accepted for a drug damaging the cell wall integrity (caspofungin is known to reduce β-glucan synthesis), it is also possible that exposure to azoles induces a salvage mechanism involving the up-regulation of chitin synthesis. Although known as a relatively minor cell wall component, chitin is thought to contribute significantly to cryptococcal wall strength and integrity [3]. Chitosan, the enzymatically deacetytaled form of chitin, helps to maintain cell integrity and is necessary for maintaining normal capsule width and retention of cell wall melanin [41]. Consistently, up-regulation was observed for *BGL2 *(2.61-fold) that encodes the glucantransferase (also termed glucosyltransferase) Bgl2, a major cell wall constituent described in a wide range of yeast species.

### Effect of FLC on genes involved in cell stress and virulence

We found that FLC induced the expression of several genes involved in oxidative-stress response (Table 1, *cell stress*). One of these genes, *GRE2*, was induced 3.54-fold, consistent with the previous observation that transcripts from *GRE2 *and other stress-induced genes (*YDR453C *and *SOD2*) were increased in *S. cerevisiae *exposed to azoles [28]. Interestingly, loss of Gre2 is impairing tolerance to ergosterol biosynthesis disrupting agents (i.e. clotrimazole and ketoconazole), further supporting an association between *GRE2 *and ergosterol metabolism [42]. *YHB1 *that encodes a flavo-haemoglobin able to detoxify nitric oxide in *C. albicans *and *C. neoformans *was down-regulated 2.32-fold in our study, which is opposed to its established relevance *in vivo *[43]. A strong reduction in the expression of *FHB1 *(the *C. neoformans *ortholog of *YHB1*) was also observed during growth of *C. neoformans *at 37°C compared to 25°C, indicating that regulation of this gene or its product at the posttranslational level may occur in response to environmental changes [44]. In contrast, *CTA1 *encoding catalase in *S. cerevisiae *was induced (2.81-fold) by FLC exposure. Together with *TSA3 *(2.09-fold) encoding thiol-specific antioxidant protein 3 (Table 1, *cell stress*) and other responsive genes with oxidoreductase activity (Table 1, *oxidoreduction*), these genes may function in response to oxidative stress. Accordingly, the stress-related gene encoding Ssa1 was also up-regulated (2.48-fold). This *C. neoformans *protein (Hsp70 family member) acts *in vivo *as transcriptional co-activator of laccase [45] and is important for the production of melanin, which is a free-radical scavenger playing a protective role in stress resistance [17].

The *C. neoformans *polysaccharide capsule is a complex structure that is required for virulence [46,47]. Interestingly, the capsule-associated gene *CAS3 *[48] was found to be up-regulated (12.16-fold) upon exposure to the drug (Table 1, *capsule synthesis*). This gene encodes a protein belonging to a seven-member protein family that includes Cap64. Treatment with FLC did not significantly change expression of the essential capsule-producing genes, *CAP10*, *CAP59*, *CAP60 *and *CAP64*. Since the cryptococcal cell wall is needed for the localization or attachment of known or putative virulence factors other than capsule (i.e. melanin, Plb1 and Bgl2), it could be hypothesized that FLC induces alterations in the cell wall which in turns affects the expression of these factors. An alternative hypothesis would be that FLC acts as a stress-generating molecule and triggers enhanced expression of virulence determinant(s) that enable to survive in hostile environments.

### Effect of FLC on genes involved in cellular transport

Several genes involved in small molecule transport and vesicular transport were either up- or down-regulated in response to FLC (Table 1, *transport*). These include *DUR3 *(plasma membrane transporter for urea, up-regulated by 4.78-fold), *MEP2*/*AMT2 *(ammonium permease, up-regulated by 3.78-fold) and *AQY1 *(aquaporin water channel, up-regulated by 2.73-fold), which all belong to the group of *C. neoformans *genes regulated by osmotic stress [49]. It is possible that defects in the plasma membrane resulting from inhibition of ergosterol biosynthesis by FLC affects transport of small molecules through the membrane. Analysis of the H99 genome sequence [16] predicted 54 ATP-Binding Cassette (ABC) transporters and 159 major facilitator superfamily (MFS) transporters, suggesting wide transport capabilities of this environmental yeast [50]. However, we found only two *S. cerevisiae *transporter homologues with significant increased expression. One is *PDR15 *that is a member of the ABC transporter subfamily exporting antifungals and other xenobiotics in fungi [51]. The other gene is *ATR1 *that encodes a multidrug resistance transport protein belonging to the MFS class of transporters. *ATR1 *expression was recently shown to be upregulated by boron and several stress conditions [52]. To date, Afr1 (encoded by *AFR1*; also termed CneAfr1) and CneMdr1 are the only two efflux pumps associated with antifungal drug resistance in *C. neoformans *[50]. Since Afr1 is the major efflux pump mediating azole resistance in *C. neoformans *[11,15], the absence of altered *AFR1 *expression could be expected. Not surprisingly, we noticed downregulated expression (2.35-fold) of *FLR1 *(for fluconazole resistance) encoding a known MFS multidrug transporter in yeast, that is able to confer resistance to a wide range of dissimilar drugs and other chemicals [53]. This may suggest that both *AFR1 *and *FLR1 *do not participate to the short-term stress induced by FLC in *C. neoformans*.

### Effect of FLC on the susceptibility to cell wall inhibitors

**Figure 3:**
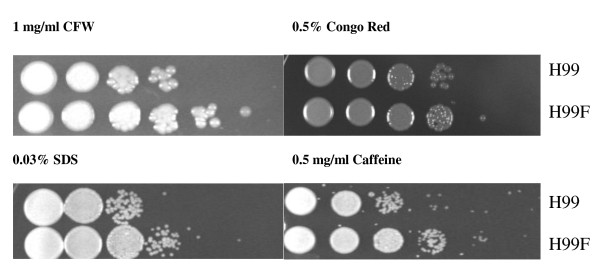
**Cell wall integrity assays with H99 *C. neoformans *cells left untreated (H99) or exposed to FLC (H99F) at a sub-MIC concentration of 10 mg/l for 90 min at 30°C**. Cells were grown at the same temperature for 48 h on YEPD supplemented with calcofluor white (CFW), Congo red, sodium dodecyl sulphate (SDS) and caffeine. Aliquots of cells were applied onto the agar surface with 10-fold serial dilutions.

It was demonstrated that compounds interfering with normal cell wall formation (Congo red, calcofluor white, SDS and caffeine) affect growth of *C. neoformans *strains with altered cell wall integrity [27]. For instance, several deletion strains for genes involved in the *PKC1 *signal transduction pathway were found to be sensitive to SDS and Congo red and to a lesser extent caffeine. To test the hypothesis that FLC treatment might induce cell wall stress, we analyzed H99 cells for susceptibility to the cell wall perturbing agents, before and after the cells were exposed for 90 min to FLC at sub-MIC concentration (10 mg/l) at 30°C. Phenotypes of H99 cells on cell wall inhibitor plates are shown in Figure 3. The FLC pre-treated H99 cells were slightly more resistant to all four cell wall inhibitors as compared to untreated cells. These findings are consistent with expression changes of cell wall associated genes identified in our microarray analysis. Particularly, since calcofluor white (which binds to chitin) disrupts the cell wall and Congo red (which binds to β-glucans) interferes with the cell wall biogenesis [27], the altered regulation of genes involved in the chitin (*CHS2 *and *CHS7*) and glucan (*BGL2*) synthesis may explain the phenotype of decreased susceptibility to cell wall stress exhibited by FLC-exposed cells. Similar results were obtained when H99 cells were pre-treated with FLC at 37°C (see Additional file 2).

### Effect of FLC on the susceptibility to H_2_O_2_

**Figure 4:**
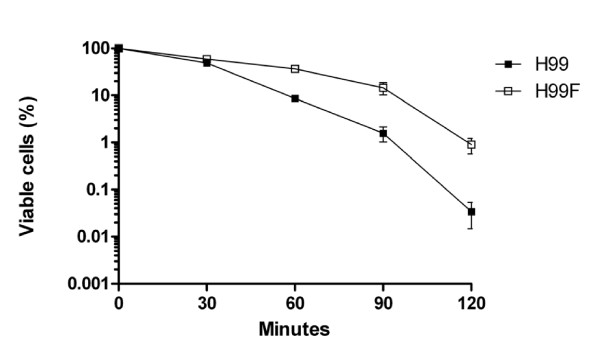
**Survival of *C. neoformans *after oxidative treatment**. Exponentially growing cells were left untreated (H99) or exposed to 10 mg/l FLC (H99F) for 90 min at 30°C and then challenged with 20 mM H_2_O_2 _for 2 h. Aliquots were harvested at given time points and cell viability performed as described in Methods. Plotted values are means of three experiments

Because a number of FLC-responsive transcriptional changes was found to affect genes involved in the oxidative stress response (i.e. *CTA1*, *GRE2*), it seemed reasonable to examine whether FLC at sub-inhibitory concentrations could induce oxidative stress resistance *in vitro*. For this purpose, exponentially growing H99 cells that were treated with 10 mg/l FLC for 90 min were subjected to an additional challenge with 20 mM H_2_O_2_. The viable cells were next quantified on YEPD plates after 0.5, 1, 1.5 and 2 h of additional growth. As shown in Figure 4, while untreated cells showed a high degree of cell death, cells treated with FLC exhibited gained more viability upon oxidative exposure at the endpoints of 1, 1.5 and 2 h. Similar results were obtained when H99 cells were pre-treated with FLC at 37°C (see Additional file 3). These findings indicate that FLC exposure is able to generate protection against oxidative stress *in vitro*, possibly as a result of a transcriptional adaptive response.

## Conclusions

Although exposure to azoles has been already investigated in several other fungal species and the transcriptional profile of differentially expressed genes was obtained using a single FLC concentration and time point, our study reveals several interesting findings. First, we demonstrated that short-term exposure of *C. neoformans *to FLC resulted in a complex altered gene expression profile. These genes included not only genes commonly responding to diverse environmental stresses, such as oxidative and drug stresses, but also genes encoding virulence factors (i.e. Plb1, Sre1 and capsule). Second, we corroborated the potential of genome-wide transcriptional analyses to envisage alternative therapeutic strategies for cryptococcosis. Apart from ergosterol and its biosynthesis, there are yet few other targets to be exploited in anticryptococcal therapy. Therefore, elucidation of molecular processes underlying the physiological responses of cryptococcal cells to FLC could serve not only to identify novel treatment approaches but also to potentiate the inhibitory effects of existing azole drugs. Our findings show that the phenomena described can apply to the *in vivo *situation, i.e. during azole maintenance therapy in the host, but transcriptional analyses using different growth conditions of H99 cells, mimicking stress conditions encountered during a human meningeal infection, may reveal new fields to pursue for anticryptococcal therapy.

## Authors' contributions

MS, DS and BP designed the study; ARF and SF carried out the experimental work; ARF, EDC and RT analysed the data; ARF and BP wrote the manuscript. GF and DS corrected the manuscript. All the authors read and approved the final manuscript.

## Supplementary Material

Additional file 1 **Table A1 Primers and fluorescent probes used in qRT-PCR**. Contains Table A1 showing the qRT-PCR primers and probes. Click here for file

Additional file 2 **Figure A1 Cell wall integrity assays with H99 *C. neoformans *cells left untreated (H99) or exposed to FLC (H99F) at a sub-MIC concentration of 10 mg/l for 90 min at 37°C.** Cells were grown at the same temperature for 48 h on YEPD supplemented with calcofluor white (CFW), Congo red, sodium dodecyl sulphate (SDS) and caffeine. Aliquots of cells were applied onto the agar surface with 10-fold serial dilutions. Contains Figure A1 showing the results of cell wall inhibitors susceptibility assays for H99 cells pre-treated with FLC at 37°C. Click here for file

Additional file 3 **Figure A2 Survival of *C. neoformans *after oxidative treatment.** Exponentially growing cells were left untreated (H99) or exposed to 10 mg/l FLC (H99F) for 90 min at 37°C and then challenged with 20 mM H_2_O_2 _for 2 h. Aliquots were harvested at given time points and cell viability performed as described in Methods. Plotted values are means of three experiments. Contains Figure A2 showing the results of H_2_O_2 _susceptibility assays for H99 cells pre-treated with FLC at 37°C. Click here for file
