# Supplementary material for: Genome-wide expression profiling of the response to short-term exposure to fluconazole in Cryptococcus neoformans serotype A
Source: BMC Microbiol. 2011 May 11;11:97. doi: 10.1186/1471-2180-11-97 (PMC3119188; doi:10.1186/1471-2180-11-97)
Supplement: Additional file 1 — Table A1 Primers and fluorescent probes used in qRT-PCR. Contains Table A1 showing the qRT-PCR primers and probes. [file 1471-2180-11-97-S1.DOC]

| Primer or probe | Gene (Accession number) or BROAD ID (CNAG_*****) | Sequence | Gene location (5’-3’) |
| --- | --- | --- | --- |
| Act-1 | *ACT1* (U10867) | ACACTGTCCCCATTTACGAAGG | 482-503 |
| Act-2 | *ACT1* (U10867) | CGGCAGAAGTGGTGAAGAGG | 594-613 |
| Actpr | *ACT1* (U10867) | TexasRed-CTCCCTTCCCCACGCTATCCTCCG-BHQ2 | 507-530 |
| Afr1-1 | *AFR1* (AJ318062) | TCAAAACGAATACCGATTCTACCG | 2175-2198 |
| Afr1-2 | *AFR1* (AJ318062) | CGCCACCAGACGAGTACAAA | 2295-2314 |
| Afr1pr | *AFR1* (AJ318062) | 6FAM-TGCTGCCTTTGCCCTCGCTGCC-TAMRA | 2202-2223 |
| 747-1 | 00747 | CCCCACACCCAAGGCTCTC | 123-141 |
| 747-2 | 00747 | GAGCGGGAGAGTCAACCATCT | 275-295 |
| 747pr | 00747 | 6FAM-CTGCCTTCTCCGCCGCCGAA-TAMRA | 143-162 |
| 1858-1 | 01858 | TTGATGGAGAACGGCGGTATC | 553-573 |
| 1858-2 | 01858 | ACCTATATTTAAACCTTTGCGGTCAT | 704-729 |
| 1858pr | 01858 | 6FAM-CACTTGGTTCATTGCTGGCTCCGA-TAMRA | 671-694 |
| 2048-1 | 02048 | CCGGACTTGGTTCGTCTACG | 303-322 |
| 2048-2 | 02048 | TCCTTGGAAGGACCAGTCTCG | 552-572 |
| 2048pr | 02048 | 6FAM-CAACTGAGACTCGTCCACTTCGGCA-TAMRA | 525-549 |
| 2226-1 | 02226 | GGTTCAACGTCAGTAATCCAAGTG | 335-358 |
| 2226-2 | 02226 | ATAAATGCCTTGCTTGAGAAGAAAGAT | 592-618 |
| 2226pr | 02226 | 6FAM-TCTCCTGACCGACTCCTGGCGACG-TAMRA | 568-589 |
| 3007-1 | 03007 | AGCCTCCTTCCCACGCTATG | 170-189 |
| 3007-2 | 03007 | CTCAGCCTGAGCCCTGGC | 289-306 |
| 3007pr | 03007 | 6FAM-TGCCTCTTAGCCTCCTCTCGGTCC-TAMRA | 264-287 |
| 3204-1 | 03204 | CTACCCTACCACGGACACTGTAT | 69-91 |
| 3204-2 | 03204 | CGTATGATTCCCTGAGAGCAAACT | 187-210 |
| 3204pr | 03204 | 6FAM-ACAACACCGTCTCGCTCAACTGGA-TAMRA | 101-124 |
| 4632-1 | 04632 | CCTACAAGCACCGAAACTACTTCA | 496-519 |
| 4632-2 | 04632 | CAGGCTGCGAGAAGATTGGAC | 680-700 |
| 4632pr | 04632 | 6FAM-TACCGCCTGCTCGTGTGATTGACC-TAMRA | 653-676 |
| 3433-1 | 03433 | TTCCCCGAAACGCTTCTCATG | 74-94 |
| 3433-2 | 03433 | AAGGTGGAACTGAGGCTGACA | 267-287 |
| 3433pr | 03433 | 6FAM-CCAGTGCGGCTCCTCGTCTTCC-TAMRA | 96-117 |
| 5264-1 | 05264 | TGCCTGTTCTTTCAAACCTCATCT | 2-25 |
| 5264-2 | 05264 | TGATAGATAGACCTTGATCGCCATTC | 70-95 |
| 5264pr | 05264 | 6FAM-CCATTCCTTGCCATCGCTCACGC-TAMRA | 34-56 |
| 5602-1 | 05602 | CGCTGAGAGGGCTGGTCT | 75-92 |
| 5602-2 | 05602 | CGTACCTGTACTTGCCAGAGATTAA | 346-370 |
| 5602pr | 05602 | 6FAM-CTGTGGACAAGATGCTCGCCGCT-TAMRA | 101-123 |

Abbreviations: 6FAM, 6-carboxyfluorescein; TAMRA, 6-carboxy-*N,N,N’,N’-*tetramethylrhodamine; Texas Red, trademark product from Molecular Probes; BHQ2, Black Hole Quencer 2.
