# Supplementary figures and images for: Genome-wide expression profiling of the response to short-term exposure to fluconazole in Cryptococcus neoformans serotype A
Source: BMC Microbiol. 2011 May 11;11:97. doi: 10.1186/1471-2180-11-97 (PMC3119188; doi:10.1186/1471-2180-11-97)

**1 mg/ml CFW 0.5% Congo Red**

**
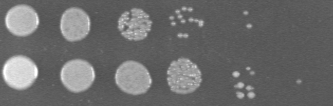

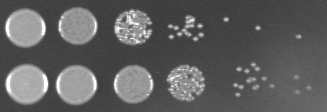
**

H99

H99F

**0.03% SDS 0.5 mg/ml Caffeine**

**
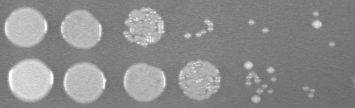

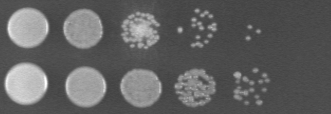
**

H99

H99F

Supplement: Additional file 2 — Figure A1 Cell wall integrity assays with H99 C. neoformans cells left untreated (H99) or exposed to FLC (H99F) at a sub-MIC concentration of 10 mg/l for 90 min at 37°C. Cells were grown at the same temperature for 48 h on YEPD supplemented with calcofluor white (CFW), Congo red, sodium dodecyl sulphate (SDS) and caffeine. Aliquots of cells were applied onto the agar surface with 10-fold serial dilutions. Contains Figure A1 showing the results of cell wall inhibitors susceptibility assays for H99 cells pre-treated with FLC at 37°C. [file 1471-2180-11-97-S2.DOC]

**
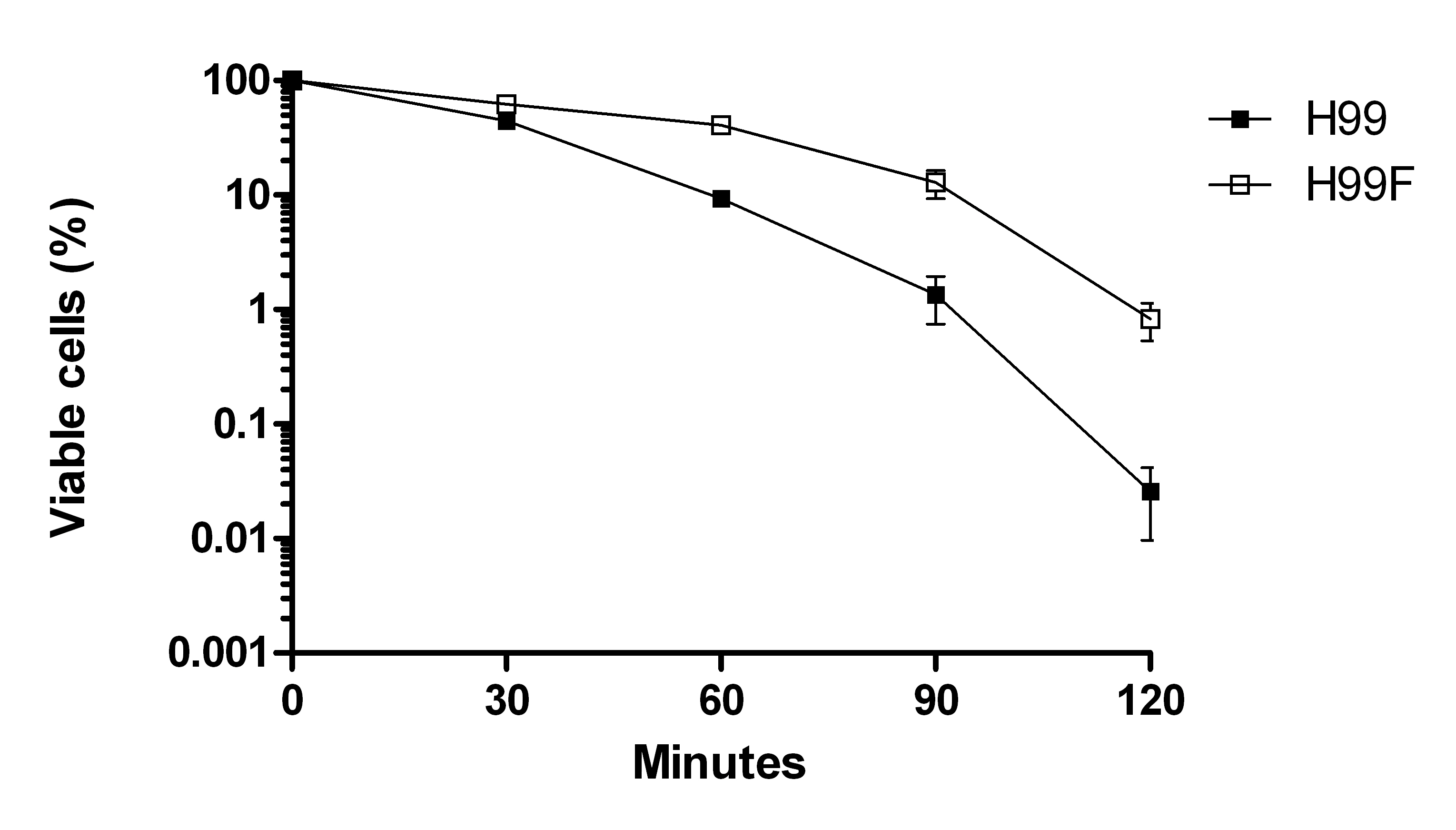
**

Supplement: Additional file 3 — Figure A2 Survival of C. neoformans after oxidative treatment. Exponentially growing cells were left untreated (H99) or exposed to 10 mg/l FLC (H99F) for 90 min at 37°C and then challenged with 20 mM H2O2 for 2 h. Aliquots were harvested at given time points and cell viability performed as described in Methods. Plotted values are means of three experiments. Contains Figure A2 showing the results of H2O2 susceptibility assays for H99 cells pre-treated with FLC at 37°C. [file 1471-2180-11-97-S3.DOC]
